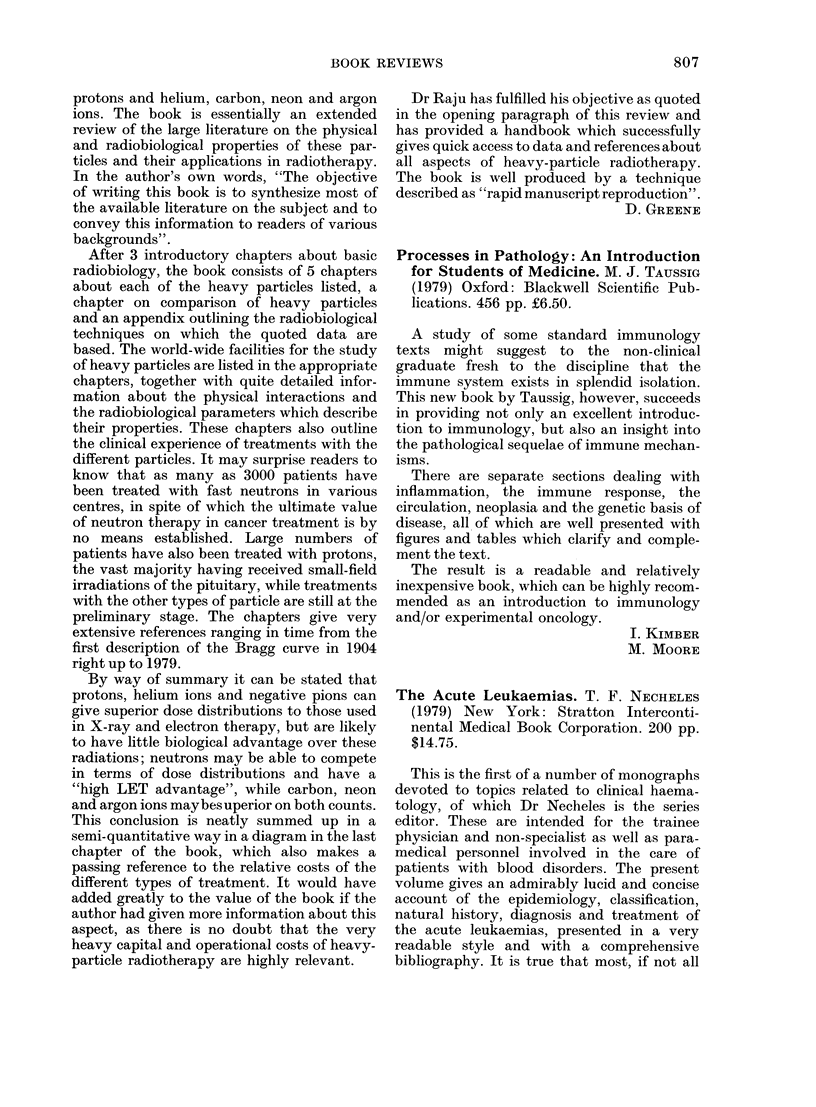# Processes in Pathology: An Introduction for Students of Medicine

**Published:** 1980-11

**Authors:** I. Kimber, M. Moore


**Processes in Pathology: An Introduction for Students of Medicine.** M. J. Taussig (1979) Oxford: Blackwell Scientific Publications. 456 pp. £6.50.

A study of some standard immunology texts might suggest to the non-clinical graduate fresh to the discipline that the immune system exists in splendid isolation. This new book by Taussig, however, succeeds in providing not only an excellent introduction to immunology, but also an insight into the pathological sequelae of immune mechanisms.

There are separate sections dealing with inflammation, the immune response, the circulation, neoplasia and the genetic basis of disease, all of which are well presented with figures and tables which clarify and complement the text.

The result is a readable and relatively inexpensive book, which can be highly recommended as an introduction to immunology and/or experimental oncology.

I. Kimber
M. Moore